# INTERGENERATIONAL CONNECTIONS: TURNING POINTS IN COLLEGE STUDENT ATTITUDES ABOUT OLDER ADULTS AND AGING

**DOI:** 10.1093/geroni/igac059.2700

**Published:** 2022-12-20

**Authors:** Wendy Watson, Sandra Faulkner, Madison Pollino, Jaclyn Shatterly, Charlie Stelle

**Affiliations:** Bowling Green State University, Bowling Green, Ohio, United States; Bowling Green State University, Bowling Green, Ohio, United States; Bowling Green State University, Bowling Green, Ohio, United States; University of Alabama, Tuscaloosa, Alabama, United States; Bowling Green State University, Bowling Green, Ohio, United States

## Abstract

Intergenerational Connections was a semester-long community-based engagement project in which thirty-four undergraduate students enrolled in a relational communication course met via Zoom with older adults in a long-term care facility for four dialogues about close relationships. The project used a Critical Interpersonal and Family Communication Pedagogy approach to have students critically engage with stereotypes and expectations about older adults through class readings, discussion, and interaction. The students wrote reflections after each dialogue and a final reflection expressing their expectations and experiences of interacting with an older adult. We analyzed the reflections using a turning point analysis and found two turning points related to Perceptions of Aging and Developmental Changes and Understanding and Expectations of Relationships. Students recognized stereotypes they held about older adults and aging and how engaging with an older adult dispelled many of those assumptions. Students were surprised by how much they had in common with their older adult partner. They learned about relationships through their dialogues with their partner and found many “words of wisdom” they wanted to incorporate into their relationships. In their final papers, students reflected on being advocates for older adults and how this project helped them understand that they can have conversations with older adults and not be afraid that they will not be able to connect. This paper will discuss how this community engagement project served to reframe aging for these young adults, and students’ recognition of the role that older adults play and the value that their involvement brings to society.

